# Is asthma a vanishing disease? A study to forecast the burden of asthma in 2022

**DOI:** 10.1186/1471-2458-13-254

**Published:** 2013-03-21

**Authors:** Teresa To, Sanja Stanojevic, Rachel Feldman, Rahim Moineddin, Eshetu G Atenafu, Jun Guan, Andrea S Gershon

**Affiliations:** 1 Child Health Evaluative Sciences, The Hospital for Sick Children, Toronto, Ontario M5G1X8, Canada; 2 Institute for Clinical Evaluative Sciences, Ontario M4N 3M5, Canada; 3 University of Toronto, Toronto, Ontario, M5S 1A1, Canada; 4 University Health Network, Toronto, Ontario, M5G 2C4, Canada

## Abstract

**Background:**

Recent evidence regarding temporal trends of asthma burden has not been consistent, with some countries reporting decreases in prevalence of asthma. In Ontario, the province in Canada with the highest population, the prevalence of asthma rose at a rate of 0.5% per year between 1996 and 2005. These estimates were based on population-based health services use data spanning more than a decade and provide a powerful source to forecast the trends of asthma burden. The objective of this study was to use observed population trends data of asthma incidence and prevalence to forecast future disease burden.

**Methods:**

The Ontario Asthma Surveillance Information System (OASIS) used health administrative databases to identify and track all individuals in the province with asthma. Individuals with asthma identified between April 1, 1996 and March 31, 2010 were included. Exponential smoothing models were applied to annual data to project incidence to the year 2022, prevalence was estimated by applying the cumulative projected incidence to the projected population.

**Results:**

While asthma incidence is falling, the absolute number of prevalent cases will continue to rise. We projected that almost 1 in 8 individuals in Ontario will have asthma by the year 2022, suggesting that asthma will continue to be a major burden on individuals and the health care system.

**Conclusions:**

These projections will help inform health care planners and decision-makers regarding resource allocation to optimize asthma outcomes.

## Background

In 2004, the Global Initiative of Asthma estimated that more than 300 million people worldwide were affected with asthma [1]. In the 1980s and 1990s, many countries reported significant increases in asthma prevalence, with more recent studies suggesting that asthma prevalence may have reached a plateau or may even be decreasing [2]. These trends seem to be dependent on a country’s asthma prevalence, whereby high prevalence countries reported decreases [2]. Conversely, in North America, both Canada and the United States reported an increase in the prevalence of asthma [3,4]. Inconsistencies in findings may be attributed to differences in study designs and data collected from different sources using varying definitions and time points [5]. An understanding of trends of disease prevalence and estimates of future disease burden can inform health policy makers regarding resource allocation in order to optimize health outcomes.

In the province of Ontario, which has a population of 13 million (one third of Canada’s population), the prevalence of asthma was estimated to be 13.3% in 2005, representing a rise of 0.5% per year since 1996 [3]. Ontario has a diverse and multi-cultural population, with several large urban centres as well as remote rural regions. Previous estimates of asthma in Ontario were based on a validated asthma definition derived from population-based health services use data [3]. These data span more than a decade and provide a powerful population-based source to forecast the trends of asthma incidence and prevalence. In this study, we aim to describe trends in asthma incidence and prevalence to forecast future disease burden based on the observed trends.

## Methods

### Data sources

Ontario has a universal, single-payer health-care system that covers all physician and hospital services. Data were available from three health administrative databases: 1) The Ontario Health Insurance Plan Database contains information on all fee-for-service billings for physician services rendered as well as emergency department visits in Ontario, including a diagnosis, 2) The Canadian Institute for Health Information Discharge Abstract Database records the primary diagnosis and up to 15 secondary diagnoses for all patients discharged from acute-care hospitals prior to 2002 and up to 25 secondary diagnoses in 2002 and later years, and 3) The Ontario Registered Persons Database includes information on gender, birth date, residence postal code, and, if applicable, date of death.

We linked these databases together on an individual level using an encrypted unique Ontario health card number given to all Ontario residents. Such linkage allows for protection of the identities of individuals while examining their health services use across health administrative databases. These databases are housed at the Institute for Clinical Evaluative Sciences in Ontario.

### Asthma case definition

The Ontario Asthma Surveillance Information System (OASIS) used the above mentioned databases to create a population-based longitudinal surveillance system which identifies and tracks individuals living with asthma [3,6,7]. Individuals with asthma were identified as those who had at least two asthma outpatient claims in two consecutive years or at least one hospitalization for asthma. This health administrative data case definition for asthma has previously been validated and has been shown to have 89% sensitivity and 72% specificity in children (under 18 years old) and 83.8% sensitivity and 76.5% specificity in adults aged 18 years or over, with a low overall false negative rate of under 2% and a false positive rate of 13.3% [8,9]. OASIS includes all individuals with asthma identified using this case definition between April 1, 1996 and March 31, 2010. Since asthma in early childhood can be misdiagnosed, and up to 50% of asthma in children remits, [10] we applied an exclusion criterion such that those with an asthma diagnosis before 5 years of age and did not have a subsequent asthma health service claim were excluded from this study. To minimize the potential for misclassifying prevalent cases as incident cases, we included a ‘look back’ period, whereby we set a minimum asthma-free observation period of 5 years prior to incidence date. If an individual had a claim in the previous 5 years, it was assumed that they were a prevalent case, not an incident case. Details regarding the identification of asthma incident and prevalence cases have been previously published [3,8].

### Measures of the burden of asthma

The asthma measures were presented as incidence (per 1,000 population) and prevalence (per 100 population) rates, stratified by sex, rural residence (communities with less than 10,000 people), and the Ontario Marginalization Index (ON-Marg) [11]. The ON-Marg is a census and geographically based index derived to show differences in marginalization between areas and to understand inequalities in various measures of health and social well-being in populations or geographical areas. This index includes four dimensions, including material deprivation (no high school graduation, lone parent families, government transfers, unemployment, low income, homes needing major repairs), dependency (seniors, ratio of population ages 0–14 and 65+ to population ages 15–64, labor force participation), residential instability (living alone, youth, persons per dwelling, apartments, married, owner-occupied house, residential mobility in past 5 years) and ethnic concentration (recent immigrants and visible minorities). The index applies to small, relatively stable population between 2,500 and 8,000 living with similar economic and social conditions. In these analyses we used the deprivation domain as a proxy measure of socio economic status, expressed in quintiles, with Q1 being the least and Q5 the most deprived populations.

### Statistical analysis

We applied double exponential smoothing methods [12] to annual data and projected incidence to the year 2022. All series were initially inspected for trend and seasonality (Cyclical Component); in this case the seasonal component did not apply. We evaluated each time series independently and chose the most appropriate model based on three diagnostic criteria: independent residuals, stationary time series and the Ljung-Box test. [13]. The final best model was selected primarily based on the agreement with assumptions of the models and the goodness-of-fit criteria. Analyses were conducted using the *forecast* package in the statistical program R [14]. The assumptions underlying the population projections (regarding fertility, immigration and mortality, interprovincial migration and non-permanent residents) are developed from various data sources: population estimates, vital statistics and administrative files. These models are based on several assumptions. 1) The observed trends during the past 14 years will reflect future trends; 2) there will be no major changes in the determinants, treatment of asthma, severity or long term prognosis; 3) that the risk factors and triggers for asthma do not change; and 4) once an individual enters the OASIS cohort it is assumed that their asthma does not remit. To estimate the prevalence until 2022, we used the projected incidence rates and applied these to the medium projected population estimates for the province of Ontario, adding the cumulative projected incidence cases to the prevalent population in 2009. The base population for these projections was derived from the official post censal estimates on July 1, 2009, with projections established using the components method.

### Estimates of absolute burden

The projected rates obtained from the time series models were then applied to the age-specific projected Ontario population to estimate the absolute asthma burden in 2012 and 2022. The Ontario population estimates for 2012 and 2022 were obtained from the medium population projections from Statistics Canada [15]. All projected numbers were presented to the nearest thousand in order to reduce errors due to rounding.

This study was approved by the Research Ethics Board at The Hospital for Sick Children Research Institute, and the Institute for Clinical Evaluative Sciences, in Toronto, Canada.

## Results

**Figure 1:**
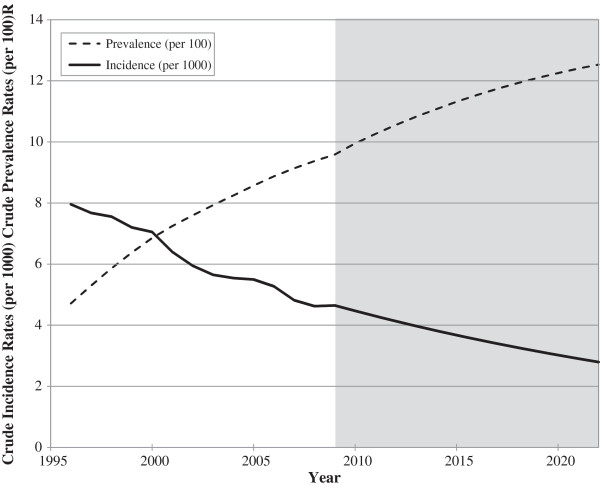
Projected incidence and prevalence rates.

Observed data from 1996 to 2009 showed a gradual decline in the newly diagnosed asthma cases in Ontario (Figure 1), and in each of the age groups (Table 1). The decreasing incidence trend was forecasted to continue and estimated to be 4.7 per 1,000 (95%CI: 3.5, 6.3) in 2022. The cumulative prevalence has been gradually increasing since 1996, and we projected this trend to continue (Figure 1, Table 1). The prevalence of asthma was forecasted to be 12.5% (95%CI: 11.3, 14.2) in 2022, increasing at a slower rate (0.2% per year) than was observed previously.

**Table 1:** Observed and forecasted asthma incidence, 1996 to 2022 in Ontario

**Observed**					**Forecasted**						
	**1996**		**2009**		**2012**			**2022**			
	**Number**	**Rate**	**Number**	**Rate**		**Rate**	(95%CI*)	**Number**	**Rate**	(95%CI*)	**ETS Model**
Ontario population	11 082 903		11 838 462								
Incidence per 1,000 population											
*Age groups in years:*											
5-9	22 101	32.7	18 092	27.9		27.9	(16.0, 48.9)	23 500	27.9	(8.7, 90.1)	M, N, N
10-19	12 606	9.3	8 444	6.0		5.8	(4.8, 7.2)	9 500	5.8	(4.6, 7.4)	A, Ad, N
20-39	20 683	6.1	9 537	2.9		2.7	(2.2, 3.3)	10 200	2.5	(2.1, 3.0)	A, A, N
40-59	15 395	5.8	10 977	3.1		3.1	(1.9, 4.8)	12 300	3.1	(1.2, 8.2)	M, A, N
60+	13 246	7.7	7 970	3.6		3.6	(2.4, 5.4)	13 600	3.6	(1.5, 8.6)	M, M, N
*Setting:*											
Rural	10 624	8.7	4 568	3.2		2.5	(2.3, 2.6)	NA	1.1	(1.0, 1.1)	A, A, N
Urban	73 294	7.8	50 429	4.8		4.3	(3.9, 4.8)	NA	3.0	(2.4, 3.7)	A, A, N
Marginalization quintiles:											A, A, N
1 (least marginalized)	15 323	5.7	15 323	4.8		4.6	(4.4, 4.9)	NA	4.5	(4.3, 4.7)	A, Ad, N
2	17 311	7.4	17 311	4.6		4.1	(3.9, 4.3)	NA	3.1	(3.0, 3.3)	M, M, N
3	16 834	8.3	16 834	4.4		3.8	(3.5, 4.2)	NA	2.3	(1.9, 2.8)	A, A, N
4	15 812	9.4	15 812	4.4		3.7	(3.3, 4.1)	NA	2.0	(1.5, 2.7)	A, A, N
5 (most marginalized)	15 337	10.5	15 337	5.0		4.2	(3.8, 4.7)	NA	2.4	(1.9, 3.0)	A, A, N

*95%CI = 95% Confidence Intervals.

Note: NA = Not applicable since no projected population data available, ETS = error, trend, seasonality, M = multiplicative, N = none, A = Additive, Ad = damped additive.

### Stratified analysis

a) Residence

Asthma incidence was consistently higher in urban populations compared to individuals living in rural regions (Table 1). However, the relative gap between urban and rural residence was forecasted to widen for asthma incidence claims in 2022.

b) Ontario Marginalization Index

In 1996 the most marginalized populations (Q5) had the highest asthma incidence (10.5 per 1,000 population), whereas in 2022 we projected that the least marginalized group will have the highest incidence of asthma (4.5 per 1,000 (95%CI: 4.3, 4.7) population), albeit a similar rate was observed in 1996.

### Absolute burden

Applying the forecasted rates to the projected Ontario population, in 2022 there will be more than 71,000 new cases of asthma and more than 1.9 million individuals living with asthma.

## Discussion

Our time series forecasting suggests that despite asthma incidence is decreasing in Ontario, asthma will continue to be a major burden on individuals and the health care system in 2022. We projected that about 1 in 8 individuals in Ontario will have asthma by the year 2022, compared to about 1 in 10 in 2009. If we applied our projected rates to the rest of Canada, we projected that there will be more than 5.5 million people (95%CI: 5.0 million, 6.2 million) living with asthma, and more than 205,000 new cases (95%CI: 152,000; 277,000) of asthma in 2022.

In our analysis we applied the same asthma definition at the population level, thereby using consistent trend data to make projections into the future. Our findings on the trends of asthma incidence and prevalence are consistent with those reported by others; the asthma prevalence in Ontario in 2009 (9.6%) was similar to that estimated in the US in 2009 (8.3%) [4]. Most recent studies have suggested evidence of a stabilization or declining asthma prevalence since the late 1990s, [16-20] however, the explanation for the apparent stabilization of asthma prevalence remains elusive. Both our observed and projected asthma incidence suggested a declining trend in Ontario, which may have contributed to the stabilizing prevalence of asthma. This stabilizing prevalence of asthma in Ontario in turn may have contributed to the decrease in asthma health services use in recent years [6,21].

Previous studies suggested that lower socioeconomic status was related to a higher asthma burden [22,23]. It may be associated with the poor affordability and accessibility of asthma medications which may still be major barriers to long-term asthma control especially in privately insured health care systems. We observed health inequalities narrowing for asthma incidence and prevalence, which have large public health implications. While individual level data pertaining to socioeconomic position and deprivation are preferable, the ON-Marg index has been demonstrated to be stable across time periods and geographic areas, and has been found to be associated with other health outcomes [11,24-28]. Furthermore, this multi-dimensional marginalization index may be a more sensitive proxy of SES, as it encompasses several elements of social well-being, not just income or education. While there is universal primary health care in Canada, there is a lack of universal insurance for medications in Ontario. Therefore, improving both the affordability and accessibility of asthma medications through public health policies and interventions is an important priority to improve asthma outcomes.

Although it is not possible to make causal inferences, our observations in asthma trends between 1996 and 2009 may reflect population level changes in risk factors and the environment which may have largely impacted the social determinants of health and thereby the most marginalized populations. For instance, smoking bans began in 1994 in Ontario with a province-wide smoking ban implemented in all pubic and work places in 2006 [29]. These policies appear to have had a direct impact on the rates of smoking; such that between 1995 and 2005 the prevalence of smoking in Ontario decreased by 18.9% [30,31]. Previous studies have shown that ambient levels of NO_2_, SO_2_, PM_2.5_ were associated with increased asthma visits in Toronto, Ontario, and that the burden of poor air quality disproportionately affected the poor [32]. Clean air initiatives have resulted in substantial improvements of air quality in the province, [33] suggesting NO_2_, CO, SO_2_, have decreased by as much as 64% since 2000 [33]. Although, these are ecological observations, they provide a contextual landscape.

Some studies in the US have reported no difference in asthma prevalence between rural and urban areas, with others reporting a higher prevalence in rural areas [34-37]. However, in Canada, a higher prevalence of asthma in urban areas has been reported in the literature [38,39]. We have also previously documented that the lifetime risk of developing asthma is higher in urban populations compared to rural populations [40]. It has been suggested that rural populations could experience lower asthma prevalence for several reasons. For example, there is less outdoor air pollution in rural communities. Secondly, growing up on farms or around animals can expose people to microbes that could lead to altered immune system development, and potentially lessen the chance of developing allergies or asthma. However, it is possible that reduced access to healthcare could lead to reduced case detection in rural communities and therefore a lower reported/estimated prevalence [41,42]. On the other hand, the higher prevalence of asthma in urban populations could be related to other risk factors such as poverty, air pollution, immigrant status, and minority status [22,23]. However, our study did not have risk factor data to measure these potential associations. Future studies could examine these determinants in greater detail.

One limitation of using health administrative databases to identify individuals with asthma is that it was based on the use of physician-diagnosed asthma only, which can be subject to misclassification bias. However, others have found that while differences in absolute values exist between health administrative data and survey data, health administrative data are consistent over time, and are thus reliable for studying asthma trends [3,43]. Another limitation is that because the study population was identified from health services use administrative data, those individuals with asthma that did not encounter the health system for either diagnosing/treating asthma would potentially be missed and were therefore unidentifiable. In Canada, a recent study conducted by Aaron et al [44] found that about one-third of individuals with physician-diagnosed asthma actually did not have asthma when objectively assessed. Their finding suggests that, in developed countries such as Canada, asthma is overdiagnosed rather than underdiagnosed.

Because asthma incidence was identified and projected from 14 years of observed data, a limitation is that it is possible for physician and patient behavior to have changed and for the diagnosis of asthma to have improved, meaning that the validation done previously would no longer be appropriate. There is also some uncertainty to the projections, demonstrated by confidence intervals, as we were limited to 14 years of observed data and projected 13 years into the future. In addition, our projections do not make assumptions regarding potential changes in asthma triggers, diagnosis or treatments in the future, and how incidence, prevalence and health services utilization may change in response to these factors. Finally, while the OASIS cohort is a comprehensive registry of asthma cases in Ontario, the nature of the cohort is such that once an individual is classified as having asthma, they remain in the prevalence pool until they die, or move from Ontario. Since we are not certain whether asthma can remit, we compared rates after applying a 20% remission rate; this figure is based on a recent study which showed 20% of the OASIS cohort did not have an asthma related visit following their index date for up to 15 years after their index date [45]. Based on these figures, we would project 1.6 million (compared with 1.9 million) people in the province of Ontario would be living with asthma in 2022. Despite the uncertainties around the projected estimates, our results have the potential to guide decision makers about health service needs related to asthma, provided they are interpreted with caution and within the context of current data. As more data become available, these projections should be updated and re-calibrated.

## Conclusion

This study applied time series methods to population-based data and demonstrated its ability to measure and project trends in asthma incidence and prevalence. These projections may help examine practice variations, monitor gaps in care across various areas and over time. Health care planners and decision-makers may use these projected estimates to identify problems, design and evaluate solutions today and for the future. While we projected a decreasing asthma incidence in the next decade, the burden of asthma remains substantial. Our findings support ongoing efforts to implement effective asthma management and patient education interventions.

## Competing interest

The authors declare that they have no competing interests.

## Authors’ contributions

To, RM, and ASG were involved in the conception, and design of the study. SS, RF, EGA and JG were involved in the analysis and interpretation. All authors contributed to the writing of the article and its revision prior to submission. All authors read and approved the final manuscript.

## Authors’ information

The Respiratory Population-based Outcomes Network: Studies and Evaluations (RESPONSE) Team (Astrid Guttmann, M. Diane Lougheed, Sharon Dell, Matthew Stanbrook, Eric Crighton, David Fisman, Susan McLimont).

## Pre-publication history

The pre-publication history for this paper can be accessed here:

http://www.biomedcentral.com/1471-2458/13/254/prepub
